# Understanding safe water‐carrying practices during pregnancy and postpartum: A mixed‐methods study in Nepal

**DOI:** 10.1111/aphw.12325

**Published:** 2021-12-03

**Authors:** Vica Marie Jelena Tomberge, Akina Shrestha, Regula Meierhofer, Jennifer Inauen

**Affiliations:** ^1^ Department of Health Psychology and Behavioral Medicine, Institute of Psychology University of Bern Bern Switzerland; ^2^ School of Medical Sciences Dhulikhel Hospital, Kathmandu University Hospital Dhulikhel Nepal; ^3^ Department of Sanitation, Water and Solid Waste for Development (SANDEC) Eawag ‐ Swiss Federal Institute of Aquatic Science and Technology Dübendorf Switzerland

**Keywords:** access to water, convergent mixed‐methods design, health action process approach, low‐income population, psychosocial determinants of health, women's health

## Abstract

Daily carrying of heavy loads of domestic water, especially during pregnancy and postpartum, bears a threat to maternal health in low‐income countries. Using an extended health action process approach (HAPA), we examined women's reasons for and psychosocial determinants of safe water‐carrying during pregnancy and postpartum. In a mixed‐methods study, trained local interviewers conducted 1001 quantitative interviews with women of reproductive age (*n* = 921 analyzed) and 21 qualitative interviews with women of reproductive age, in‐laws, and spouses in rural Nepal. We analyzed the quantitative data with generalized estimating equations to model the HAPA‐based psychosocial determinants of avoiding water‐carrying during pregnancy and postpartum. Subjective perspectives were investigated with thematic analysis. Outcome expectancies (*B* = 0.24), self‐efficacy (*B* = 0.20), and injunctive norms (*B* = 0.23) were significantly associated with the intention to avoid water‐carrying. Self‐efficacy (*B* = 0.36) and instrumental support (*B* = 0.05) are related to behavior (all *p* < 0.05). Women explained water‐carrying during pregnancy by a lack of family support, a shift of health decision‐making power to in‐laws, and low behavioral control. Overall, the necessity of water, family decision‐making structures, and low support make it difficult for women to discontinue water‐carrying. Additionally to infrastructural improvements, behavioral interventions may increase women's self‐efficacy for safe water‐carrying (e.g. reducing weight) and social support.

## INTRODUCTION

The need for water poses an emotional and physical burden on women's daily work in low‐income countries (Geere et al., 2018; Sharma & Singh, 2012). Water is needed for drinking, hygiene, and food preparation for household members and for livestock. It can involve lifting and carrying 20‐kg water containers several times per day (Geere et al., 2010). Many women in rural areas retain the responsibility for carrying water during their pregnancies (Regmi, 2007). Although reproductive organs require 6 months to heal postpartum (Romano et al., 2010), Nepali women in rural areas return to their physically demanding working routine shortly after giving birth, as little as 7‐ to 30‐day postpartum (Earth & Sthapit, 2002; Panter‐Brick, 1989).

Handling heavy items such as water containers during pregnancy and postpartum poses a range of risks for maternal and child health, including increased demands on the musculoskeletal system, increased uterine contractility, threats to fetal growth, preterm delivery, spontaneous abortion, and uterine prolapse (Darshan, 2009; Earth & Sthapit, 2002; Juhl et al., 2013; Koyuncu et al., 2021; MacDonald et al., 2013). The social consequences of prolapse are substantial and include physical and emotional isolation, low self‐esteem, inability to work, lack of economic support, and domestic violence (Darshan, 2009; Gunasekera et al., 2007). The United Nations Population Fund estimates that 10 per cent of women in Nepal suffer from uterine prolapse, and heavy lifting is one of the main causative factors (Gunasekera et al., 2007). Encouraging behaviors that prevent these physical health impacts and their psychosocial consequences might help to minimize adverse health outcomes and enhance women's quality of life.

### Safe water‐carrying

No international recommendations or specific guidelines are available for Nepal, but the American Institute for Occupational Safety and Health recommends weight limits for pregnant workers that can be acceptable to 90 per cent of healthy women and were designed as a guideline for employers (MacDonald et al., 2013; Waters et al., 2014). It recommends maximum load of 15 kg in the first half of pregnancy and up to 11 kg in the second half with an ideal posture for repetitive lifting. These maximum weight recommendations can only be considered safe for an ideal lift: infrequent two‐handed lifting of compact loads close to the body without twisting, stooping, or reaching up or forward. Because the ideal pose for lifting the object close to the body might be obstructed in the second half of pregnancy, a reduction to 7 kg may be considered. Additionally, objects should not be lifted from the ground. The recommended load is lower for specific work tasks and body conditions (MacDonald et al., 2013), for instance, when carrying water during pregnancy in hilly areas such as Nepal. Regular bending of the waist as when lifting loads from below midshin (e.g. water containers from the ground) results in a three‐times‐higher risk of preterm labor and miscarriages (Bonzini et al., 2009).

No evidence‐based recommendations are available for the postpartum period, but guidelines (Howard County General Hospital & John Hopkins Medicine, n.d.; Oxford University Hospitals, 2016) suggest that no loads heavier than the baby should be carried until 6‐week postpartum and no “very heavy loads” (not further specified) should be carried until 3‐month postpartum.

Many women in low‐income countries do not meet these safe carrying strategies (Geere et al., 2018; Sharma & Singh, 2012). They are often required to carry water, firewood for cooking, grass and leaves for animal feed, and farm produce to the house and market. Depending on the circumstances, women may fetch 15 to 40 L of water in one trip (Sharma & Singh, 2012). In Nepal, the daily routine of lifting 20‐kg water containers from the ground and sometimes carrying them uphill is continued by many women during pregnancy and shortly after giving birth (Earth & Sthapit, 2002; Geere et al., 2018).

### Explaining safe water‐carrying practices during pregnancy and postpartum

It is necessary to understand women's lifeworld and why they apply unsafe or safe carrying strategies. To date, only a small number of qualitative studies and no quantitative studies have sought to explain why women in general continue heavy work in the vulnerable periods of pregnancy and postpartum. These studies found that sociostructural factors that perpetuate unsafe working behavior during pregnancy and postpartum include economic disadvantages, social norms, and expectations about the division of labor within families and partners' lack of economic support or willingness to reduce their wives' workloads during pregnancy (Lowe et al., 2016; Mullany, 2006; Panter‐Brick, 1989). In addition, individual psychological determinants may steer water‐carrying practices. Literature on uterine prolapse indicates that one possible psychological factor may be insufficient perception of the risk of carrying heavy loads during pregnancy and postpartum (Shrestha et al., 2014).

Health behavior models such as the health action process approach (HAPA) may provide a more comprehensive understanding of the sociocognitive determinants of safe water‐carrying as a health behavior (Schwarzer, 2008). The HAPA model posits predictors that facilitate the formation of intention and the planning and realization of behavior such as safe water‐carrying. Predictors leading to intention formation include risk perception, outcome expectancies such as beliefs about the consequences of safe water‐carrying, and self‐efficacy, such as belief in the ability to perform safe water‐carrying. Predictors of behavior include intention, action planning, such as when, where, and how to safely carry water, and coping planning, which involves anticipating barriers and making alternative plans. Finally, the HAPA also foresees contextual barriers and resources such as social support that need to be considered for the performance of behavior.

The HAPA model has proven applicable to a broad range of health behaviors, populations, and cultures, including the global South (Renner et al., 2007; Zhang et al., 2018). In addition to the HAPA factors, researchers of health behavior in low‐income countries have often identified social norms as predictors of health behavior (Harter et al., 2018; Reid et al., 2010; Tumwebaze & Mosler, 2014). These include injunctive norms, which are conceptions of what significant others approve of, and descriptive norms, which are what significant others do (Cialdini et al., 1991). Hence, social norms within Nepali families, predominantly the husband and the mother‐in‐law, and communities might predict women's workload during pregnancy and postpartum (Lowe et al., 2016; Mullany, 2006; Panter‐Brick, 1989). Considering family members' perspectives on safe water‐carrying might therefore provide insight not offered by former studies.

### The present study

In summary, no studies have investigated the psychosocial determinants of safe water‐carrying practices during pregnancy and postpartum. The HAPA model's broad applicability indicates that it may provide a useful framework for predicting safe water‐carrying. This study will be the first based in theory to test the psychosocial determinants of maternal workload in low‐ and middle‐income settings. However, because the HAPA has not been applied in this setting, women and family members' perspectives should also be considered in qualitative interviews so as not to miss unexpected insights and cross‐validate quantitative findings. This mixed‐methods study aims to explain the psychosocial factors of safe water‐carrying during pregnancy and postpartum by addressing these two research questions. Quantitatively, what are the psychosocial determinants of the intention (I) and behavior (II) of safe water‐carrying? Qualitatively, how do women and their family members explain women's water‐carrying during and after pregnancy?

## METHODS

This mixed‐methods study was conducted in 2019, in five communities in the Kavre and Sindhupalanchowk districts of Nepal, which are in a typical rural low‐income region with a mixture of at‐house and off‐plot water supplies such as water sources in yards and neighborhoods and communal sources.

We used a convergent mixed‐methods design (Creswell, 2015) to investigate the reasons for unsafe water‐carrying in low‐income Nepal, which referred specifically to water‐carrying during pregnancy and postpartum. The narrative structure of qualitative data allows it to highlight women's and their family members' subjective perspectives without superimposing any theory. In contrast, the quantitative data allow a more generalizable theory‐based test of psychosocial determinants of the intention and behavior to carry water safely. We paired the results to identify areas that converged and diverged across the two methodologies (Creswell, 2015) and thus to arrive at strong conclusions about the psychosocial factors that can explain safe water‐carrying during pregnancy and postpartum. All participants provided written informed consent prior to their interviews. If during the interview a participant indicated symptoms of reproductive health problems, a free screening at the local health center was performed, and if necessary, free treatment was offered.

### Quantitative methods

#### Participants and procedures

Eight female Nepali data collectors were trained to carry out a cross‐sectional structured survey. They interviewed a random sample of approximately 200 women in each of five communities (*N* = 1001) using the random route method (Hoffmeyer‐Zlotnik, 2003). The study areas were selected because each had an outreach center of [blinded] Hospital. The data collectors presented themselves and the aim of the study when arriving at the house and started the interview when a woman in the household was willing to participate and met the selection criteria: being adult—from 16 years in Nepal—and of reproductive age, permanently residing in the community, and being involved in water‐carrying. When more than one woman met the criteria, the data collectors interviewed the woman predominantly responsible for water‐carrying.

As our main outcome focused on carrying behavior during pregnancy, we excluded 80 women (8%) that had never been pregnant. This resulted in a sample size of *n* = 921 for all analyses. The survey was conducted in Nepali and supervised by the first and second authors. To ensure participants' privacy, they were interviewed inside the house in case other adults gathered outside the house.

### Measures

The interviews assessed details of carrying behavior, women's mental and physical health, social context, psychosocial determinants of water‐carrying during and after pregnancy, and standard demographic measures. Please consult Table S1 in the supporting information for all measures and individual items, descriptive statistics, and internal consistencies. Consult Table S3 for bivariate correlations. All survey instruments were translated and back‐translated from English to Nepali and adapted to the local context in close collaboration with our Nepali partners before and after a pretest.

The target behavior of safe water‐carrying was defined as avoiding carrying water during pregnancy and 3 months postpartum. This was assessed by a self‐report behavior index that measured the water‐carrying frequency in one typical week during pregnancy and one typical week at 3 months postpartum, referring to the previous pregnancy, for example, “How often in 1 week did you carry water in the 3 months after delivery?*”*, 1 = “every day” to 5 = “no days”. The answer given was then reverse coded to represent avoidance.

The survey instruments assessing psychosocial determinants of safe water‐carrying were developed based on the theoretical framework of the HAPA model (Schwarzer, 2008) and social norms (Cialdini et al., 1991). They included risk perception, outcome expectancies, self‐efficacy, behavioral intention, instrumental support, action planning, coping planning, injunctive norm, and descriptive norm. Internal consistencies for the constructs were high, with Cronbach's alpha 0.83 < *α* < 0.91 except for the injunctive norm. At the first field site, the injunctive norm exhibited low internal consistency, but it achieved satisfactory consistency when excluding this site (*α* = 0.72). As the results did not change when excluding the site from analyses, we reported the results for all sites. Unipolar 5‐point Likert scales were used to assess psychological constructs. Data collectors used a visual scale of five dots of increasing size to visualize answer categories, which ranged from “I do not at all agree” to “I agree very much” (Harter et al., 2020).

### Data analysis

To model the psychosocial determinants of intentions and behavior of avoiding water‐carrying during pregnancy and postpartum, we performed two generalized estimating equations (GEE). GEE can estimate the parameters of a generalized linear model and serve as a flexible generalization of ordinary linear regression (Nelder & Wedderburn, 1972). The GEE accounted for the structure of the data, which was nested as households in communities (Liang & Zeger, 1986). We included the following predictors: risk perception, outcome expectancies, self‐efficacy, injunctive norm, descriptive norm, and instrumental support, as psychosocial determinants of intention (Model 1) and self‐efficacy, injunctive norm, descriptive norm, behavioral intention, instrumental support, action planning, and coping planning as psychosocial determinants of behavior (Model 2). Both models included the following control variables: age, socioeconomic status, historical pregnancy, living condition, ethnicity, education, and type of water source. We computed all analyses in IBM SPSS Statistics 24 (IBM Corp., Armonk, N.Y., USA). All variables and sample syntax can be found in the supporting information.

## QUALITATIVE METHODS

### Participants

To gain the best possible insights, we used theoretical sampling to understand all aspects of women carrying water during pregnancy and 3‐month postpartum (Glaser, 1999). The initial approach was to interview women of reproductive age and women who had a daughter‐in‐law to include the perspectives of mothers‐in‐law. The initial plan was to interview six women from different age groups in different areas in each of the five communities to cover a heterogeneous sample. After identifying some key concepts in a first round of interviews, we specifically approached daughter–mother‐in‐law and wife–husband dyads to gain a deeper understanding of family decision‐making and responsibility structures. This resulted in a total of 44 transcripts.

The final sample size for the qualitative analysis was then determined by data saturation, which was reached after analyzing 21 interviews. This final sample included five daughter–mother‐in‐law dyads (*n* = 10), three wife–husband dyads (*n* = 6), one wife–mother‐in‐law husband triad (*n* = 3), and two single women (*n* = 2). The qualitative sample therefore included 21 interviews.

### Data collection and measures

The first author, the second author, and a Nepali public health graduate student conducted the first half of the semistructured qualitative interviews together. After having assisted in four interviews, a Swiss psychology master's student conducted the second half of the interviews with the Nepali graduate student. The Nepali researchers translated the interview questions and responses simultaneously to the non‐Nepali speaking researcher. All interviewers engaged in constant reflective exchange to ensure sensitivity towards the interviewees and themselves. The team audiorecorded the interviews. The second author transcribed and translated them into English ad verbatim.

A sample guideline for the qualitative interviews can be found in Table S4 of the supporting information. We used semistructured interviews to ask explicitly about women's daily routines and family members' behaviors and attitudes during pregnancy and postpartum, and we encouraged participants to articulate the reasons for their behavior and thinking with nondirective follow‐up questions. Further, we developed a narrative storytelling task that used two pictures illustrating a fictitious scenario of a woman with a newborn child, either carrying water or not. On presenting the pictures, we asked open questions, such as “what kind of woman is she?”. We used this task to stimulate discussion about more implicit thoughts on the sociocultural norms, values, and expectations about this situation (find information on storytelling in Feldman et al., 2004).

### Data analysis

For the qualitative analyses, we used a systematic, iterative approach of inductive reasoning, by which a theory forms from the data (Charmaz & Henwood, 2017). To ensure comparability with the quantitative data, the analysis focus was set on women in reproductive age. Additional information was gained by analyzing the transcripts of family members. Transcripts were analyzed until theoretical saturation was reached and no additional insight or new codes were found. We conducted a bottom‐up thematic analysis (Braun & Clarke, 2006), which includes familiarization with all transcripts, generation of initial codes, search for themes, review of themes, definition and naming of themes, and writing up the themes analyzed. The first author first read all transcripts, familiarizing herself with the data, and writing down initial codes. She then searched for themes that represented some level of patterned response or meaning within the data set in relation to the research question. These themes and assigned codes and quotations were reviewed and discussed with the last author and the second author separately in order to validate them. The first author then identified relationships between themes, and combined them to larger themes wherever necessary. All authors then reviewed the themes and relationships again until they reached consensus. In order to validate the results, the first author examined all transcripts a second time, looking for contradictory evidence, also termed deviant cases (Anderson, 2010). The first author then wrote up the analyzed themes, including the most representative quotation in order to provide evidence to the reader. Then, the co‐authors reviewed a first report of the results and discussed feedback in light of the research questions and the reliability of the findings.

## RESULTS

### Quantitative results

The women interviewed were around 35 years old (*SD* = 9 years), half of them (45%) had an average monthly household income of 9600 NPR or less (≈80 US$), 89 per cent received less than 24,000 NPR (≈200 US$), and half (53%) of them did not pass primary school. The average number of pregnancies per interviewee was 2.9 (*SD* = 1.6). All other sample characteristics can be found in Table S2 of the supporting information.

Safe water‐carrying (avoiding carrying during previous pregnancy and postpartum) (*M* = 0.40, *SD* = 0.38) and intention of avoiding carrying (*M* = 0.63, *SD* = 0.29) was around the midscale. The mean weight carried by nonpregnant women was 20 kg (*SD* = 10 kg) per trip; women currently pregnant or postpartum (*n* = 65) carried 18 kg (*SD* = 11 kg). Some 55 per cent and 51 per cent of women reported carrying smaller quantities of water during pregnancy or postpartum respectively. Women also reported carrying other heavy loads (*M* = 36 kg, *SD* = 12 kg), 65 per cent of them daily. On average, women reported high risk perception (*M* = 0.70, *SD* = 0.33) and outcome expectancies (*M* = 0.78, *SD* = 0.25) and low‐to‐moderate self‐efficacy (*M* = 0.42, *SD* = 0.34) and injunctive norms (*M* = 0.56, *SD* = 0.25) for avoiding water‐carrying during pregnancy and postpartum. Most women (89%) received instrumental support in carrying water (see all descriptive statistics in supporting information Table S1).

As shown in Table 1, the higher the self‐efficacy, the more positive were the outcome expectancies and the more favorable the injunctive norm to avoid water‐carrying, the higher was the intention of doing so.

**TABLE 1:** Generalized estimating equations of psychosocial determinants of intention and behavior to avoid water‐carrying during pregnancy and postpartum

	Behavioral intention for safe water‐carrying					Safe water‐carrying behavior				
		95 per cent CI					95 per cent CI			
Parameter	*Estimate*	*SE*	LL	UL	*p*	*Estimate*	*SE*	LL	UL	*p*
Intercept	0.21	0.08	0.05	0.37	0.011	−0.09	0.10	−0.29	0.10	0.341
Risk perception	0.06	0.03	< 0.01	0.12	0.052	‐	‐	‐	‐	‐
Outcome expectancies	0.24	0.06	0.13	0.34	< 0.001	‐	‐	‐	‐	‐
Self‐efficacy	0.20	0.06	0.08	0.31	0.001	0.36	0.05	0.25	0.46	< 0.001
Injunctive norm	0.23	0.10	0.04	0.43	0.019	0.09	0.05	< 0.01	0.18	0.056
Descriptive norm	0.02	0.04	−0.05	0.09	0.666	0.04	0.05	−0.05	0.13	0.388
Behavioral intention	‐	‐	‐	‐	‐	0.20	0.06	0.09	0.31	< 0.001
Instrumental support	0.05	0.03	−0.02	0.11	0.160	0.05	0.02	0.01	0.10	0.031
Action planning	‐	‐	‐	‐	‐	−0.02	0.04	−0.09	0.05	0.627
Coping planning	‐	‐	‐	‐	‐	0.02	0.03	−0.04	0.07	0.575
Age	< 0.01	< 0.01	< 0.01	< 0.01	0.047	< 0.01	< 0.01	−0.01	< 0.01	0.224
Socioeconomic status^a^	< 0.01	< 0.01	< 0.01	< 0.01	0.056	< 0.01	< 0.01	< 0.01	< 0.01	< 0.001
Living without husband	−0.01	0.02	−0.04	0.03	0.719	0.03	0.01	< 0.01	0.05	0.044
Currently pregnant^b^	0.03	0.03	−0.03	0.08	0.323	−0.02	0.03	−0.08	0.03	0.421
Currently delivered^c^	−0.01	0.02	−0.05	0.03	0.663	−0.03	0.04	−0.10	0.04	0.436
Education^d^	< 0.01	< 0.01	−0.01	0.01	0.459	0.01	0.01	−0.01	0.02	0.238
Ethnicity^e^										
Brahmin	−0.02	0.01	−0.05	0.01	0.150	0.11	0.06	−0.01	0.22	0.072
Tamang	−0.04	0.03	−0.08	0.01	0.167	0.09	0.05	< 0.01	0.17	0.059
Newar	−0.04	0.03	−0.10	0.03	0.305	0.10	0.03	0.04	0.17	0.002
Chhetri	0.06	0.03	0.01	0.11	0.017	0.09	0.07	−0.04	0.22	0.156
Dalit	−0.01	0.01	−0.04	0.02	0.397	0.05	0.05	−0.05	0.15	0.323
Rai and Limbu	−0.02	0.03	−0.07	0.04	0.546	0.12	0.05	0.02	0.23	0.016
Water source^f^										
Household tap	−0.03	0.03	−0.09	0.03	0.357	0.01	0.06	−0.10	0.13	0.832
Shared tap	−0.05	0.03	−0.12	0.02	0.150	−0.02	0.04	−0.10	0.07	0.716
Community tap	−0.04	0.02	−0.08	< 0.01	0.046	−0.02	0.04	−0.11	0.06	0.592

*Note*: *N* = 921. Five communities. *Estimate =* parameter estimates. *SE* = standard error. CI = confidence interval. Probability distribution: normal, link function: identity. All *p‐*values are two‐tailed. All variables were recoded to a range between 0 and 1.

^a^ Socioeconomic status: An index (0.0–1.0) was calculated using principle component analysis (Krishnan, 2010).

^b^ Currently pregnant: Are you currently pregnant?

^c^ Currently delivered: Have you delivered in the last three months?

^d^ Education: higher values refer to a higher level of education: 0 = illiterate, 1 = informal education, 2 = preprimary, 3 = primary passed 4 = Lower secondary passed, 5 = secondary, 6 = higher secondary and above.

^e^ Ethnicity: Reference = other.

^f^ Water source: Reference = source further than village.

For behavior, the analyses showed that higher self‐efficacy and intention and more instrumental support from any family member were associated with less frequent water‐carrying during pregnancy and postpartum.

## QUALITATIVE RESULTS

The analyzed interviews included 12 women (*M* = 33 years, monthly expenses *M* = 20,791 NPR [≈ 173US$], 50% no primary education), 4 husbands (*M* = 50 years, monthly expenses *M* = 21,705 NPR [≈ 180 US$], 50% no primary education), and 5 mothers‐in‐law (*M* = 57 years, monthly expenses *M* = 9900 NPR [≈ 82US$], 100% no primary education). Further sample characteristics can be found in Table S5 of the supporting information. The perspectives of women and their family members on women's behavior and its reasons converged. Therefore, we present the quotation that best illustrates each of the themes below. More quotations for each theme and additional subthemes can be found in supporting information Table S6.

### Carrying behavior during pregnancy and postpartum

All the women interviewed indicated moderate to high risk behavior during their pregnancy and/or 3 months postpartum (see Table S5). Some women carried during pregnancy but not postpartum. Some carried throughout the whole period of pregnancy; some carried only until a certain month. In addition to water, women carried other heavy loads for agricultural purpose: *“When doing fieldwork*, *we have to carry crops*, *corn*, *and fertilizers*. *And we also carry other loads like grass and firewood*. *We have to carry*.*”* 5_daughterinlaw: 27.

Further, it became apparent that whether they carried water or not depended on women's physical ability. In cases of sickness, they would not carry. But as long as they were able to, women carried water during pregnancy and postpartum: “*I do whatever I can*, *and what I can't do I don't do*. *I'm not able to carry loads anymore*, *so my body does not feel weak*.*”* 1_woman: 31. In cases of pregnancy‐ or delivery‐related difficulties, they did not carry water: *“Even though I like carrying water*, *I can't at the moment [Note: respondent is pregnant]*. *… If I could do it*, *I would have felt nice*. *Now I need to tell others*. *If I could*, *I would have brought the water myself*.*”* 2_woman: 98.

### High risk perception but low personal vulnerability

The perception of the risks of water‐carrying in general and water‐carrying during pregnancy and postpartum was high in most family members. Risks mentioned included pain, burden to mental health, complications during pregnancy, and concerns about child health and uterine prolapse: “*It's risky*. *They may get uterine prolapse*, *bleeding*, *headache*, *and back pain*. *The effects are not known when she carries*, *but later it may cause health hazards*.*”* 6_husband: 91.

Some women considered themselves less vulnerable to developing uterine prolapse due to their good health condition or past experience. *“I have three babies*. *I don't know*, *nothing happened*. *We also carried gagris [water containers]*, *used to work a lot*, *nothing happened to us*.*”* 3_woman: 178.

### Negative and positive outcome expectancies for the avoidance of carrying during pregnancy and postpartum

Both negative and positive outcome expectancies were mentioned. The advantages of not carrying during pregnancy and postpartum mentioned were the prevention of adverse health effects, uterine prolapse specifically, and bearing a healthy child. Two women mentioned this: *“We can be protected from diseases during pregnancy if we don't have to carry loads*.*”* 12_daughter: 185; “*We shouldn't work if we want our uteruses to be safe*. *We shouldn't carry heavy loads*. *During pregnancy too*, *we shouldn't work a lot these days*.*”* 1_woman: 285.

Advantages of carrying during pregnancy and postpartum mentioned included the need to provide food and water and the belief that carrying is convenient for women's and children's health and would facilitate the delivery:

*“It's said that the more I work*, *the more my health will be good and my baby will be energetic*. *So*, *when you're pregnant*, *if you don't work then you'll have problems during the delivery*. *So*, *if I work*, *then I'll have an easy delivery*. *The more I work*, *the easier it will be for my body*.*”* 3_woman: 174.

Other women indicated that they carried because of favorable affective attitudes: “Actually, it's not obligatory [to carry in this period], but I like to do that. My heart doesn't allow me to stay and do nothing. I'm carrying out of choice.” 2_woman: 223–241. Some women reported feeling “bad” or “odd” when not able to perform the behavior: “It was hard [to carry during pregnancy], but I felt rather odd not carrying water while living here.” 8_daughterinlaw: 101.

Some women reported a low response efficacy of avoiding carrying: “*In some cases*, *even if women do nothing*. *then there is uterus prolapse*.” 7_daughterinlaw: 220.

### Belief in karma

Finally, the belief in karma that good people deserve well was also given as a reason for carrying water during pregnancy, mostly in the picture task: “*She [carrying woman] is a blessed woman*. *It can be understood just by looking who is blessed and who has bad family*.*”* 13_motherinlaw: 297. *“She might be blaming her luck and karma*.*”* 11_daughterinlaw: 121.

### Lack of options and necessity of carrying water

A lack of options was mentioned, associated with necessity: *“Without water there's no food*. *So we decided [that] after delivery she carried water after 10‐15 days*. *What can be done? We needed water*.*”* 6_husband: 75–81. Obligation was also mentioned: *“Even though we feel it to be difficult*, *we need to [carry water]*. *We have no option*.*”* 1_woman: 229.

The availability of household taps and vehicles was mentioned as one reason why women carried loads during pregnancy and postpartum or not (Table S6). However, household taps were not always reliable and not all water was carried from there. Some families needed to switch to other sources during the dry season; one husband said: “*Six months [in dry season]*, *we have to carry water from a well rather far away*.*”* 12_husband: 21.

### Social influence and decision‐making

Family members and their attitudes were frequently mentioned as contributing to women's water‐carrying during pregnancy and postpartum. When getting married, women might be expected to work in their in‐laws house: *“When I was 15 years old*, *I got married and then started lifting heavy loads*.*”* 1_woman: 71;*“Daughters‐in‐law do household work*, *agricultural work and take care of the cattle*, […]. *I expect them to help more*, *take care of the cattle*, *cook food*, *cut grass*, *and collect firewood*.” 5_motherinlaw: 59–61.

Injunctive norms were also mentioned. Avoiding work was disapproved, for instance, when women did not assume their familial responsibility to bring water: “*She [mother in law] didn't say much to me*, *but she was angry when I didn't work*. *… They [family members] sometimes behave rudely*, *saying that I'm taking rest [postpartum] while they have to work*.*”* 11_daughterinlaw: 173–181. Conversely, other women mentioned that family members approved of their avoiding carrying during pregnancy and postpartum: “*Others also said that we should rest two months*, *so I did*.*”* 10_daugtherinlaw: 170, or that they were without any expectations: *“She [mother‐in‐law] is happy with whatever I do*. *She is happy with me*.*”* 5_daughterinlaw: 79.

In the picture task, some women expressed approval and admiration of the women who carried postpartum for fulfilling their responsibilities in this period: *“I think she [carrying woman] is brave and daring*.*”* 11_daughterinlaw: 111. *“She [carrying woman] must be a very good woman*. *At the time when she has to rest … she is carrying water*.*”* 9_wife: 109.

Some women reported that their in‐laws or husbands told them what to do or not to do: *“If things are like that [that I am pregnant] then they [in‐laws] need to agree [not to carry]*. *Sometimes if they don't agree*, *then I need to work*. *That's it*. *Mostly*, *they agree*.*”* 1_woman: 257. One husband said: *“We didn't let her carry heavy loads*, *loads from far away*; *we didn't let her carry water or firewood*. *We only let her do easy work*.*”* 12_husband: 122.

Descriptive norms about carrying during pregnancy and postpartum were mentioned by all family members: “*In the villages*, *we carry even when we're pregnant*.*”* 8_daughterinlaw: 87. On the other hand, descriptive norms about avoiding carrying were mentioned in the picture task: *“People don't work for one month when they are in the postpartum phase*.*”* 14_woman: 130.

### Social support as a resource for avoiding water‐carrying

Women received instrumental, informational, and emotional support from their social environment. Instrumental support was mostly provided by and expected from family members: *“[After delivery] my husband and father in law helped [to carry water]*. *… After I gave birth and was bleeding for some time*, *my sister's daughters helped me a lot*.*”* 12_wife: 167–173. This support was described as reciprocal within families: *“When I'm unable*, *then my mother‐in‐law takes care*, *and when she's unable*, *I help out*. *It's just natural*.*”* 5_daughterinlaw: 43. In turn, help with water‐carrying from people outside the family was not common: *“No*, *no one [other villagers] brings water for us*, *and we don't have to help others*.*”* 6_wife: 97.

However, support from family members was not always consistent or was insufficient for women to rest completely during pregnancy and postpartum (Table S6). For example, one woman said: “*He [the husband] has to go to work*, *and there's nobody who works in the home*, *so I need to do it*. *Sometimes if the gagri [water container] is big*, *then he (husband) helps*; *the smaller one I need to bring myself*.*”* 3_woman: 73. One woman said that her husband did not help at all: “*He doesn't work at all and doesn't do any chores*; *I have to do it all myself*.*”* 8_motherinlaw: 83.

Family members gave informational support to the woman with advice about how to behave during pregnancy and postpartum and in case of sickness *“Everyone in the family helped and supported her [my wife*, *when she was pregnant]*. *They gave her advice about childbirth*.*”* 11_fatherinlaw: 125; “*He [my husband] will say not to carry heavy loads*, *exercise*, *eat nutritious food*; *he says things like that*.*”* 2_woman: 297. Additionally, people outside the family might give informational support, for instance about health risks: “*I would tell [other women] not to carry too much load*.*”* 10_motherinlaw: 170.

Finally, women reported receiving emotional support during pregnancy and postpartum from friends, neighbors, and their biological mother (see additional quotations in Table S6): *“I had a buffalo*, *and goats*, *even though my child was very small*. *During that time [postpartum] the mother‐in ‐aw and father‐in ‐aw never helped*. *They really made my life difficult*. *But other friends were sympathetic to me and […] the kids*. *They used to say ‘you faced lots of problems*, *your mother‐in‐law could take care of your children’*.*”* 8_motherinlaw: 160.

### Making plans and overcoming barriers

There were strategies for carrying less weight with smaller vessels and making fewer trips during pregnancy and postpartum: “*[During pregnancy I carried] a little less but three times a day*.*” 5_daughterinlaw: 138–139*; *“I told her [daughter in law] to do less work*. *She brought water one or two times per day [during pregnancy]*. *[On normal days she carried] five or six times a day*. 5_motherinlaw: 135–137. In contrast to this, other women mentioned that they carried the same quantity at the same frequency. Regardless of pregnancy, several strategies were mentioned as safe carrying techniques (see quotations in Table S6): body postures, using helping tools, and reducing loads, for instance, by using a small water container of 10 L rather than the usual size of 20 L.

Women mentioned difficulties that may arise and plans to overcome these. If other people told them to carry water, one strategy mentioned was not listening to others. If no one helped to carry, they might also ask for help or pay someone: *“If no family members will [help postpartum]*, *then I can use money and make people help me by paying them*. *I can pay people to carry water*, *grass and other loads*.*”* 11_daughterinlaw: 193. Additionally, structural improvement was mentioned as a strategy for reducing carrying: *“They can use pipes to bring water inside and use sacks and busses to bring loads instead of carrying [during pregnancy and postpartum]*.*”* 10_daugtherinlaw: 225.

## DISCUSSION

**FIGURE 1:**
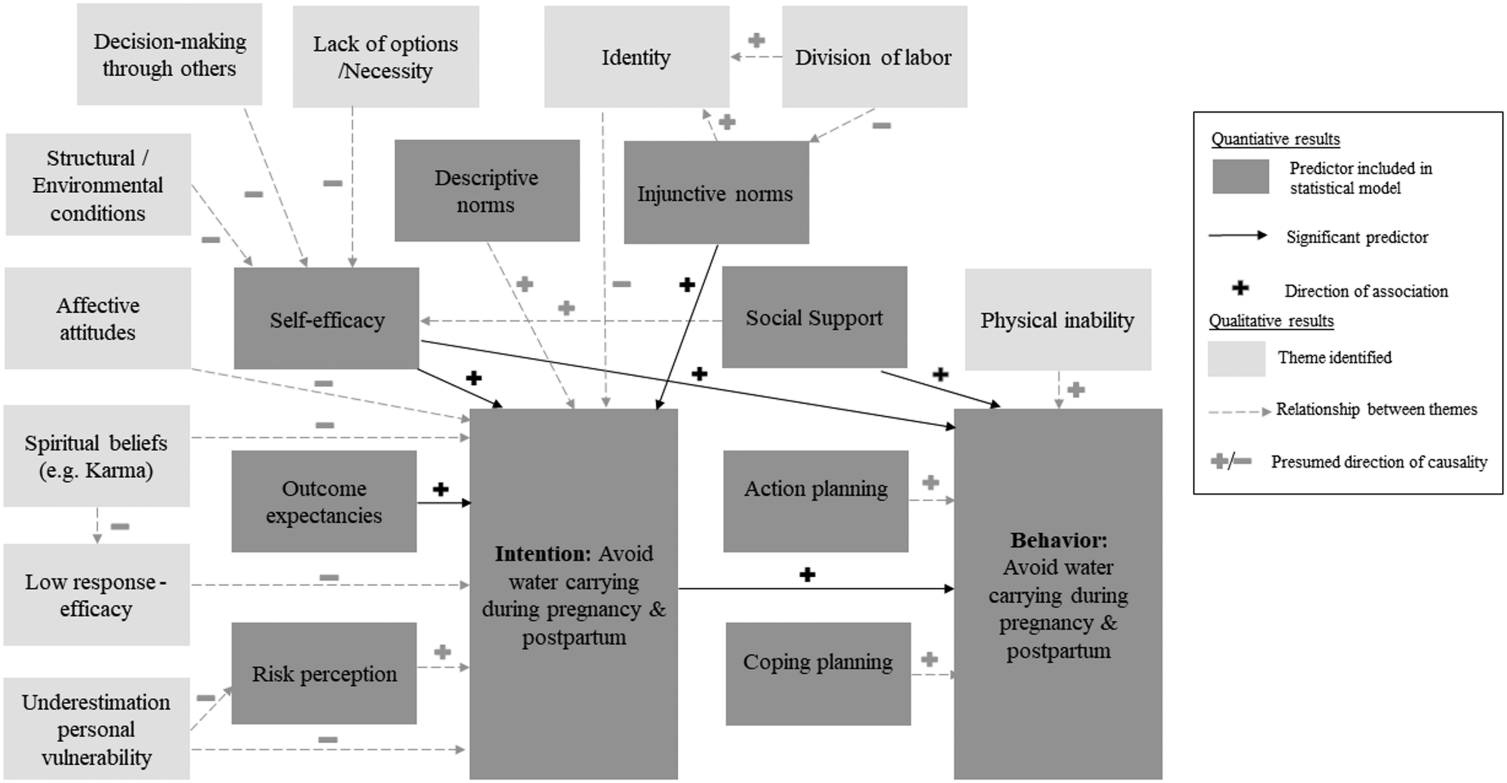
Summary of the quantitative and qualitative results indicating psychosocial determinants of safe water‐carrying. *Note*: The quantitative findings on the theory‐based predictors are dark‐gray colored. Black arrows indicate significant associations, and signs indicate their directions with safe water‐carrying derived from GEE. The themes found in the qualitative analysis are represented by light‐gray squares. Gray arrows and signs indicate causal relationships assumed based on the qualitative findings

Combining a theory‐based approach with women's and their family members' subjective experiences, this mixed‐methods study provided convergent evidence of the determinants of safe water‐carrying practices in a low‐income setting. Consistent with previous research, we found that at least half of the women in rural areas of Nepal engaged in high‐risk behavior by carrying water and other heavy loads during pregnancy and postpartum (Earth & Sthapit, 2002; Geere et al., 2018). As indicated by GEE, outcome expectancies, self‐efficacy, and injunctive norms were associated with the intention to use safe water‐carrying strategies. In turn, behavioral intention, self‐efficacy, and instrumental social support were associated with safe water‐carrying behavior. The qualitative findings corroborate these psychosocial factors and supplement them by in‐depth insights into how women explain their water‐carrying practices. Figure 1 shows a summary of the determinants of safe water‐carrying identified by quantitative and qualitative findings.

### Women's motivation to engage in safe water‐carrying

Even though respondents associated a large range of different health risks with water‐carrying, our results indicate that knowledge of these risks does not keep women from carrying water during pregnancy and postpartum. This converges with evidence from meta‐analyses that risk perception is the weakest predictor of health behavior (e.g. Zhang et al., 2018). Underestimating personal risk because of good health condition or previously escaping the consequences of risky behavior is a possible reason identified in our study. This optimism bias is a common phenomenon in social psychology and has also been documented for other water‐related health risks, such as arsenic (Flanagan et al., 2015).

Going beyond the outcome expectancies of safe water‐carrying assessed in the quantitative analysis, the qualitative data yielded the insight that women also expected positive outcomes for carrying during pregnancy and postpartum, such as a stronger body, an easier delivery, and positive feelings associated with water‐carrying. This is in line with previous research indicating that affective attitudes are important predictors of health behavior and can have independent and more powerful effects on intentions and behavior than instrumental attitudes (Lawton et al., 2009). Water‐carrying is a source of emotional distress for many women worldwide (Sultana, 2011), yet some women may like carrying water during pregnancy and postpartum, perhaps because they enjoy social interaction at the tap or the opportunity to spend time outside the household (Sultana, 2011). Future quantitative research should consider these themes as additional outcome expectancies.

Another motivational factor, injunctive norms were significantly associated with the intention of safe carrying. Women may be motivated to assume their responsibilities within the family's division of labor even during pregnancy, because studies have found that traditional Nepali families approve of equal engagement in day labor without regard to age, sex, physical fitness, or maternal status (Panter‐Brick, 1989). The finding that some women felt “odd” when not carrying water might support the interpretation that the task of water‐carrying is part of their social identity (Moran & Sussman, 2014; Verplanken & Orbell, 2003). Social norms and identity might therefore be considered to be additional factors motivating behavior that are not included in the HAPA model (Freivogel & Visschers, 2020).

The qualitative results also indicated that safe water‐carrying had low response efficacy because some women believed they might suffer from uterine prolapse even when resting after pregnancy. Further, beliefs about karma may entail the idea that health outcomes are predominantly related to people's deeds and God's will instead of attributing the outcomes to health behavior (Colditz, 2015). Such spiritual beliefs may be also considered in the HAPA model as an integral part of outcome expectancies for intention formation. Recommendations for interventions include recognizing these beliefs and incorporating them in health behavior intervention planning for Buddhist and Hindu communities (Colditz, 2015).

### Sociostructural barriers and resources related to safe water‐carrying behavior

Although the motivational factors mentioned above can explain women's intention to carry water safely, we identified various sociostructural barriers that can prevent even motivated women from pursuing their goal in the volitional phase of behavior (Schwarzer, 2008). The quantitative results indicated that self‐efficacy was the weakest of all psychosocial determinants but the strongest predictor of intention and behavior. A self‐efficacious person responds confidently with strategies to implement an intended behavior and overcome barriers through coping planning (Schwarzer, 2016). Many women had specific plans for reducing unsafe water‐carrying, which included reducing the weight to be carried. However, our qualitative results indicated that lack of social support, family members' decisions and expectations, and poor household water access might diminish self‐efficacy in implementing these plans. This is in line with other studies which found that sociostructural barriers, especially social expectations, can reduce the decision‐making control of women over their health behavior in low‐resource populations (Lewis et al., 2015; Wight et al., 2012; Wingood & DiClemente, 2000; World Health Organization, 2010).

Conversely, our quantitative and qualitative findings converged to show that family members, mostly husbands and mothers‐in‐law, provided valuable social support to overcoming barriers. The qualitative results also indicated that emotional and informational support was given by people outside the household as well but only rarely help with carrying.

Women's social networks can therefore both support and impede their health behavior (Hohl et al., 2018, 2019). Other studies on prevention of uterine prolapse in Nepal insist on increasing the involvement of their social environment to help women adopt low‐risk behaviors (Earth & Sthapit, 2002; Radl et al., 2012). A more generalizable insight into the influence of family members on safe water‐carrying (e.g. mothers‐in‐law attitudes on their daughters‐in‐law) requires quantitative dyadic approaches to health behavior change models such as the HAPA. Such approaches have been shown to be valuable in encouraging health behavior change in romantic couples (Berli et al., 2018).

We identified the frequent absence of the husband and other family members as a barrier for support. This emphasizes that interventions aiming to prevent adverse health effects of carrying during pregnancy and postpartum should consider single women as an especially vulnerable target group.

The necessity of water and other agricultural supplies for daily life further highlights the need for infrastructural improvements in the study area. Examples of such improvements include bringing the water and other loads closer to the households by constructing piped water supplies to household taps and roof ropeways and cable cars. However, even when household taps exist, it is moments of lifting and lowering heavy loads that pose the highest risks for maternal and child health (MacDonald et al., 2013; Waters et al., 2014). Behavior change to safe‐lifting strategies, such as applying specific lifting postures and reducing loads (MacDonald et al., 2013; Waters et al., 2014), might therefore complement infrastructural improvements.

### Strengths and limitations

This mixed‐methods study is the first to triangulate generalizable individual data (*N* = 921) regarding maternal water‐carrying strategies with comparative in‐depth information about family members' attitudes in a rural low‐resource population. The results showed that health behavior models such as the HAPA are applicable in settings such as this, especially when complemented by social influences.

Even though measures and transcripts were carefully translated using forward‐ and back‐translation, divergent interpretations by the participants or the researchers may have biased the findings. This divergence may limit the generalizability of the qualitative results in particular. Some results are likely culture specific (e.g. beliefs about karma), and the findings may not be readily transferable to women from other cultures. It is also important to note that the study is limited by its cross‐sectional design. Therefore, no conclusions may be drawn from the quantitative data about the causality of relationships between psychosocial factors and safe water‐carrying intentions and behavior. Although some of the qualitative results strengthen causal interpretations, longitudinal studies and particularly randomized controlled trials are needed as a next step.

Further, the data were self‐reported, and we cannot exclude the possibility of social desirability, especially when discussing sensitive family dynamics. Finally, future research should investigate carrying behavior during pregnancy and postpartum as separate determinants. Although our quantitative data indicated a strong correlation between the two behaviors (*r* = 0.7), the qualitative data revealed that these may differ.

In the present manuscript, we focused on carrying frequency. However, some women also mentioned reducing weight of load during pregnancy. We therefore recommend to include this variable in future research. To do so, specific national guidelines on weight limits during pregnancy and postpartum are needed, considering that western guidelines might not be fully transferrable to the Nepali context.

### Conclusions

In conclusion, health psychology theory and methods proved useful in investigating water‐carrying practices in a low‐income setting, provided that they are carefully adapted to the local context. We found strong convergent evidence that women in rural Nepal are aware of the risk factors entailed in carrying water during pregnancy and postpartum. However, low self‐efficacy in avoiding risky behavior due to the necessity of providing water, family decision‐making structures, and low support make it difficult for women to discontinue water‐carrying during this vulnerable period. Although structural improvements will likely facilitate safe water‐carrying, behavior change interventions are needed. These might focus on increasing women's self‐efficacy and behavioral intentions at the individual level and improving the social acceptance and support from family and community of not carrying loads during pregnancy and postpartum. Future randomized controlled trials are needed to test whether such interventions can promote safe water‐carrying practices, prevent uterine prolapse, and improve women's health and well‐being.

## CONFLICT OF INTEREST

None.

## ETHICS STATEMENT

Ethical clearance was given by the Ethical Review Committee of the Health Research Council Nepal (Reg No. 517/2019) and the Ethical Review Board of the University of Bern, Switzerland (2019‐10‐00003).

## Supporting information


**Table S1:** Items and descriptive statistics.
**Table S2:** Sample characteristics for quantitative data.
**Table S3:** Bivariate correlations between all constructs.
**Table S4.** Sample guideline for qualitative interviews.
**Table S5:** Sample characteristics and carrying behavior derived from qualitative interviews.
**Table S6.** Additional quotations for qualitative themes.
**Table S7.** SPSS SYNTAX to model two generalized estimating equations (GEE).

## Data Availability

The quantitative data described in this article and the qualititve transcipts are not openly available only to ensure confidentiality and anonymity of participants. We went through the informed consent sheets again and realized that we may not make data openly available. “I know that all personal data will be kept confidential and will not be shared with anyone other than members of our survey team. I do agree that the researchers involved in this study, public authorities, and the members of the ethical review boards in Nepal and in Switzerland while keeping confidentiality can access original data. I was informed that any information about me will have an identification number on it instead of my name. I can request the deletion of my personal data until the link between my name and the data will be deleted.”
