# Supplementary material for: Understanding safe water‐carrying practices during pregnancy and postpartum: A mixed‐methods study in Nepal
Source: Appl Psychol Health Well Being. 2021 Dec 3;14(2):691–711. doi: 10.1111/aphw.12325 (PMC9300039; doi:10.1111/aphw.12325)
Supplement: Supplementary file 1 — Table S1: Items and descriptive statistics. Table S2: Sample characteristics for quantitative data. Table S3: Bivariate correlations between all constructs. Table S4. Sample guideline for qualitative interviews. Table S5: Sample characteristics and carrying behavior derived from qualitative interviews. Table S6. Additional quotations for qualitative themes. Table S7. SPSS SYNTAX to model two generalized estimating equations (GEE). [file APHW-14-691-s001.docx]

**Understanding safe water-carrying practices during pregnancy and postpartum:**

**A mixed-methods study in Nepal**

**SUPPORTING INFORMATION**

[**TABLE S1**: Items and descriptive statistics. 1](#_Toc86837737)

[**TABLE S2**: Sample characteristics for quantitative data. 3](#_Toc86837738)

[**TABLE S3**: Bivariate correlations between all constructs. 5](#_Toc86837739)

[**S4.** Sample guideline for qualitative interviews. 7](#_Toc86837740)

[**TABLE S5**: Sample characteristics and carrying behavior derived from qualitative interviews 18](#_Toc86837741)

[**TABLE S6.** Additional quotations for qualitative themes. 19](#_Toc86837742)

[**S7.** SPSS SYNTAX to model two generalized estimating equations (GEE). 25](#_Toc86837743)

# **TABLE S1**: Items and descriptive statistics.

| Concept | Items | *M* /*f* | *SD* / *f*% | Cronbach’s alpha |
| --- | --- | --- | --- | --- |
| Safe water-carrying^1^ | How often in one week did you carry water during pregnancy? / How often in one week did you carry water in the three months after delivery?*, 1 = every day to 5 = no days* | 0.40 | 0.38 | 0.78 |
| Risk perception^1^ | Compared to other women with the same age, how much higher or lower are your chances of getting uterine prolapse? *-2 = lower* to *2 = higher^1^* | 0.70 | 0.33 | 1 item |
| Outcome expectancies^1^ | How much do you agree to the following statements *1=agree not at all to 5 = agree very much*  I would protect myself from negative health impact if I avoided water-carrying during pregnancy and after delivery  I would prevent uterine prolapse if I avoided water carrying during pregnancy | 0.78 | 0.25 | 0.84 |
| Self-efficacy^1,2^ | How sure are you that you can always avoid to carry your water during and after pregnancy?  How sure are you that you can always avoid to carry your water during and after pregnancy even if it might be difficult for your household to have enough water?  How sure are you that you can always avoid to carry your water during and after pregnancy even if someone in your family (e.g. your mother-in-law) told you to? | 0.42 | 0.34 | 0.91 |
| Intention^1,2^ | How strongly do you intend to always avoid water carrying during and after pregnancy?  How strongly do you intend to reduce water carrying during and after pregnancy?  How frequently do you intend to avoid carrying water during and after pregnancy? | 0.63 | 0.29 | 0.83 |
| Injunctive norm^1,2^ | How much would people who are important to you approve if you carry water during and after pregnancy? (reverse coded)  How much would other people in your community approve if you avoided water carrying during and after pregnancy?  How much would people who are important to you approve if you avoided water carrying during and after pregnancy? | 0.56 | 0.25 | 0.66 (0.72^3^) |
| Descriptive norm | How many women in your community carry water during and after pregnancy?*1 = Almost nobody (0%)* to *5 = almost all of them (100%)*^2^ | 0.57 | 0.27 | 1 item |
| Instrumental support | Who will assure your household's water supply in case you cannot carry enough water during and after pregnancy? (multiple answers possible) *1 = Husband; Mother-in-law; Father in law; Daughters/Sons; Other family member; Other men of the community; Other women of the community; Other; 0 = Nobody* | 823 | 89% |  |
|  | Husband | 538 | 58% |  |
|  | Mother-in-law | 259 | 28% |  |
|  | Father in law | 97 | 11% |  |
|  | Daughters | 141 | 15% |  |
|  | Sons | 103 | 11% |  |
|  | Other family member | 191 | 21% |  |
|  | Other men of the community | 16 | 2% |  |
|  | Other women of the community | 10 | 1% |  |
|  | Other | 46 | 5% |  |
|  | Nobody | 98 | 11% |  |
| Action planning | Can you tell me what you can do to avoid carrying water during and after pregnancy? (multiple answers possible, no options prompted) *1 = Ask for help; Carry less water; Tell other people I don't want to carry water; Buy water; other specific plan*; *0 = No plan* | 783 | 85% |  |
|  | Ask for help | 462 | 50% |  |
|  | Carry less water | 549 | 60% |  |
|  | Tell other people I don't want to carry water | 112 | 12% |  |
|  | Buy water | 5 | 1% |  |
|  | Other *(most frequently named: connecting pipe from source to home)* | 23 | 3% |  |
|  | No plan | 138 | 15% |  |
| Coping planning | Which difficulties may arise that can prevent you from avoiding carrying water during and after pregnancy? |  |  |  |
|  | How can you overcome difficulties that prevent you from avoiding to carry water during and after pregnancy? ^4^ (multiple answers possible, no options prompted)*1= Ask someone for help; Tell that I do not want to carry water; other specific coping plan; 0 = No plan* | 557 | 60% |  |
|  | Ask someone for help | 468 | 51% |  |
|  | Tell that I don‘t want to carry water | 64 | 7% |  |
|  | Other specific coping plan | 100 | 11% |  |
|  | No plan | 364 | 40% |  |

*Note:* *n* = 921, *M* = Mean, *SD* = Standard deviation, *f* = frequency. ^1^All continuous items were recoded to a range between 0 to 1; ^2^*0 = not at all* to *5 = very much;* ^3^without first field site; ^4^ This question was also asked when respondents did not mention any specific difficulty.

# **TABLE S2**: Sample characteristics for quantitative data.

| Concept | Options | | *M* /*f* | *SD* / *f*% |
| --- | --- | --- | --- | --- |
| Age |  | | 34.5 | 8.6 |
| Education | Illiterate | | 176 | 19% |
|  | Informal education | | 257 | 28% |
|  | Pre-primary | | 51 | 6% |
|  | Primary passed | | 138 | 15% |
|  | Lower secondary passed | | 94 | 10% |
|  | Secondary | | 103 | 11% |
|  | Higher secondary and above | | 102 | 11% |
| Socioeconomic status^1^ | |  | 0.5 | 0.1 |
|  | | What kind of fuel do you use MAINLY for cooking? |  |  |
|  | | Wood ( = 0) | 573 | 62% |
|  | | Gas ( = 1) | 348 | 38% |
|  | | What is the average expenditure of your family per month? |  |  |
|  | | Less than 2400 Nepali Rupees (~ 20 US$) | 77 | 8% |
|  | | 2500 to 4800 Nepali Rupees (~ 40 US$) | 200 | 22% |
|  | | 4900 to 9600 Nepali Rupees (~ 80 US$) | 269 | 29% |
|  | | 9700 to 24000 Nepali Rupees (~ 200 US$) | 232 | 25% |
|  | | >25000 Nepali Rupees (~ 208 US$) | 105 | 11% |
|  | | Are you the owner of your house? ( yes = 1) | 902 | 98% |
|  | | How much land does your family own? | 54.8 | 73.6 |
|  | | How many rooms does your house have? | 2.9 | 1.5 |
|  | | Does anyone from your household own any of these items? Radio, TV, solar panel, mobile phone, bicycle, motor bike, car, fridge, watch (sum) | 3.2 | 2.0 |
| Involved in economic activities |  | | 653 | 71% |
|  | Agriculture | | 521 | 57% |
|  | Other (trading, government service, daily laborer) | | 132 | 13% |
| Religion |  | |  |  |
|  | Hinduism | | 654 | 71 % |
|  | Buddhism | | 255 | 27% |
|  | Others | | 12 | 1 % |
| Marital status |  | |  |  |
|  | Married | | 887 | 96% |
|  | Widowed | | 33 | 4% |
| Living without husband | 1 = yes | | 198 | 21% |
| Number of pregnancies |  | | 2.9 | 1.6 |
| Currently pregnant | 1 = yes | | 41 | 4% |
| Delivered last 3 months | 1 = yes | | 24 | 2% |
| Ethnicity | Brahmin | | 283 | 31% |
|  | Tamang | | 275 | 30% |
|  | Newar | | 55 | 6% |
|  | Chhetri | | 53 | 6% |
|  | Dalit | | 115 | 13% |
|  | Rai and Limbu | | 126 | 14% |
|  | Others | | 14 | 2% |
| Main source in rainy season | Private tap, hand pump or tank delivery in the court or in the house | | 448 | 49% |
|  | Shared tap, hand pump, tank delivery or surface water in the close neighborhood | | 232 | 25% |
|  | Community tap, hand pump, tanker delivery or surface water in the village | | 210 | 23% |
|  | Water source further away than the village | | 31 | 3% |

*Note:* *n* = 921, *M* = Mean, *SD* = Standard deviation, *f* = frequency. ^1^An index was calculated using principle component analysis (Krishnan, 2010).

# **TABLE S3**: Bivariate correlations between all constructs.

|  |  | 1 | 2 | 3 | 4 | 5 | 6 | 7 | 8 | 9 | 10 | 11 | 12 | 13 | 14 | 15 | 16 | 17 | 18 | 19 | 20 | 21 | 22 |
| --- | --- | --- | --- | --- | --- | --- | --- | --- | --- | --- | --- | --- | --- | --- | --- | --- | --- | --- | --- | --- | --- | --- | --- |
| 1 | Water-carrying behavior^1^ |  |  |  |  |  |  |  |  |  |  |  |  |  |  |  |  |  |  |  |  |  |  |
| 2 | Risk perception | 0.10** |  |  |  |  |  |  |  |  |  |  |  |  |  |  |  |  |  |  |  |  |  |
| 3 | Outcome expectancies | 0.19** | 0.59** |  |  |  |  |  |  |  |  |  |  |  |  |  |  |  |  |  |  |  |  |
| 4 | Self-efficacy | 0.46** | 0.19** | 0.29** |  |  |  |  |  |  |  |  |  |  |  |  |  |  |  |  |  |  |  |
| 5 | Intention | 0.36** | 0.28** | 0.39** | 0.47** |  |  |  |  |  |  |  |  |  |  |  |  |  |  |  |  |  |  |
| 6 | Action planning | 0.19** | 0.16** | 0.23** | 0.34** | 0.33** |  |  |  |  |  |  |  |  |  |  |  |  |  |  |  |  |  |
| 7 | Coping planning | 0.15** | 0.07* | 0.12** | 0.27** | 0.21** | 0.31** |  |  |  |  |  |  |  |  |  |  |  |  |  |  |  |  |
| 8 | Instrumental support | 0.17** | -0.05 | 0.04 | 0.23** | 0.17** | 0.12** | 0.07* |  |  |  |  |  |  |  |  |  |  |  |  |  |  |  |
| 9 | Injunctive norm^1^ | 0.36** | 0.16** | 0.26** | 0.63** | 0.45** | 0.29** | 0.27** | 0.22** |  |  |  |  |  |  |  |  |  |  |  |  |  |  |
| 10 | Descriptive norm | 0.06 | 0.09** | 0.04 | 0.06 | 0.06 | -0.04 | < 0.01 | -0.05 | 0.12** |  |  |  |  |  |  |  |  |  |  |  |  |  |
| 11 | Age | -0.15** | -0.02 | -0.08* | -0.14** | -0.17** | -0.10** | -0.01 | -0.16** | -0.14** | 0.08* |  |  |  |  |  |  |  |  |  |  |  |  |
| 12 | Socioeconomic status | 0.15** | 0.17** | 0.18** | 0.16** | 0.17** | 0.11** | 0.05 | 0.10** | 0.14** | -0.05 | -0.01 |  |  |  |  |  |  |  |  |  |  |  |
| 13 | Living without husband | 0.01 | -0.01 | -0.02 | -0.02 | -0.03 | 0.01 | -0.05 | -0.08* | -0.05 | 0.04 | 0.02 | -0.06 |  |  |  |  |  |  |  |  |  |  |
| 14 | Currently pregnant | 0.04 | -0.02 | -0.01 | 0.06 | 0.06 | < 0.01 | -0.01 | 0.06 | 0.06 | 0.02 | -0.26** | -0.03 | -0.06 |  |  |  |  |  |  |  |  |  |
| 15 | Currently delivered | 0.06 | 0.01 | -0.03 | 0.10** | 0.04 | -0.01 | -0.01 | 0.06 | 0.08* | 0.01 | -0.18** | 0.02 | 0.05 | -0.04 |  |  |  |  |  |  |  |  |
|  | Education | 0.20** | 0.18** | 0.16** | 0.22** | 0.21** | 0.15** | -0.01 | 0.15** | 0.15** | -0.02 | -0.61** | 0.19** | 0.05 | 0.15** | 0.17** |  |  |  |  |  |  |  |
| 16 | Brahmin | 0.04 | 0.16** | 0.09** | < 0.01 | 0.02 | -0.04 | -0.02 | -0.02 | -0.07* | 0.06 | 0.12** | 0.25** | 0.04 | -0.04 | 0.05 | 0.12** |  |  |  |  |  |  |
| 17 | Tamang | -0.02 | -0.10** | -0.11** | 0.04 | -0.06 | 0.04 | 0.06 | -0.01 | 0.05 | -0.05 | .08* | -0.06 | 0.01 | -0.03 | 0.03 | -0.12** | -0.44** |  |  |  |  |  |
| 18 | Newar | 0.02 | -0.04 | 0.02 | 0.01 | < 0.01 | 0.02 | 0.03 | 0.04 | 0.06 | -0.01 | 0.03 | 0.05 | < 0.01 | -0.03 | -0.04 | -0.02 | -0.17** | -0.16** |  |  |  |  |
| 19 | Chhetri | 0.02 | 0.04 | 0.06 | 0.03 | 0.09** | 0.05 | -0.03 | -0.05 | < 0.01 | -0.01 | 0.04 | 0.01 | 0.04 | 0.01 | -0.04 | 0.02 | -0.17** | -0.16** | -0.06 |  |  |  |
| 20 | Dalit | -0.071* | -0.05 | 0.03 | -0.06 | -0.01 | -0.05 | -0.02 | < 0.01 | -0.05 | -0.04 | -0.12** | -0.22** | -0.02 | < 0.01 | -0.04 | -0.06 | -0.25** | -0.25** | -0.10** | -0.09** |  |  |
| 21 | Rai and Limbu | 0.02 | -0.03 | -0.06 | -0.04 | -0.01 | < 0.01 | -0.05 | 0.05 | 0.01 | 0.03 | -0.19** | -0.11** | -0.06 | 0.11** | -0.01 | 0.04 | -0.27** | -0.26** | -0.10** | -0.10** | -0.15** |  |
| 22 | Others | -0.01 | < 0.01 | 0.01 | 0.03 | 0.04 | 0.03 | 0.03 | 0.01 | 0.07* | 0.02 | -0.06 | 0.05 | -0.04 | -0.03 | -0.02 | 0.02 | -0.08* | -0.08* | -0.03 | -0.03 | -0.05 | -0.05 |

*Note*: Significance levels: * p < 0.05, * * p < 0.01 (two-tailed). ^1^ How often in one week do you carry water during pregnancy? / How often in one week do you carry water in the three months after delivery?*, 1 = no days to 5 = every day*

# **S4.** Sample guideline for qualitative interviews.

**Leading questions are bold,** Follow-up questions (optional) in italics

Introduction and inclusion criterion

Hello. My name is ___and this is ___. I am working with [blinded] on a study on women’s health and their quality of life here in Kavre District.

Date:

ID of respondent:

Part 1a: Free Exploration Women’s daily routine

**Please tell me about your daily routine.**

| Carrying behavior |
| --- |
| *What do you like in your day?*  *What don’t you like?*  *Why?* |
| Feelings and thoughts about tasks in daily routine |
| **Are there any tasks that make your body feel weak or hurting?** |
| **Are there any tasks that give you mental stress? Why?** |

Part 2: Free Exploration Consequences Water Carrying

**First, we would like you to tell us a bit more about your task to carry water.**

| Carrying behavior |
| --- |
| **How much water do you carry and how often?**  *Why?*  *Do you change this quantity/ amount for any reason?* |
| **Which other loads do you carry?**   - *When?* - *How much, how often?* |
| **Do you prefer carrying water or carrying other loads? Why?** |
| **When did you start carrying water?**  *Were your carrying tasks different when you were a girl?* |
| **Did your carrying tasks change when you moved from your parents house to your in-laws house?** |
| *Psychological consequences water carrying* |
| **Can you describe your thoughts and feelings about daily water carrying?** |
| *Are there times you cannot go for water carrying?*   - *Why? Tell me about the incident(s)?* - *How do you feel when this happens?* |
| *Physical Consequences Water* |
| **How does your body feel when you carry water?**   - *During water carrying* - *After water carrying* - *How does this affect your life?* |
| *Social Consequences* |
| **Please tell me more about how water carrying is organized in your village** |
| **What would you do in the time you usually carry water if you had not to carry water?**   - **Now** - **When you were a girl** |
| *If their carrying behavior changed (e.g. in birth home they didn’t carry water or they have a household tap now etc.):* |
| **Did anything in your life change since you carry water/ do not carry water anymore?** |
| *Intervention acceptance: Safe carrying technique* |
| **How can you change your carrying routine to prevent yourself from health impacts?** |
| **What do you think would be a safe carrying technique?**  regarding to   - amount/ - body position/ - tools? |
| **Would you like to implement the safe carrying technique in your daily life? Why?/ Why not?** |
| **What would you need to implement the safe carrying technique in your life?** |
| **How do you think you could learn it?** |
| **Do you think it is feasible for you to carry only small gagris (10-15 liter) or less?**  **Why? Why?** |

Part 3 Free Exploration Mother-in-law

| *General Family structures* |
| --- |
| **Please tell me about your household members and their role in your family.** |
| **Please tell me about your mother-in-law** |
| *Responsibilities in laws* |
| **What do you expect from her? What does she expect from you?** |
| *Relationship quality* |
| **How would you describe your relationship with your mother-in-law?** |
| *How can you tell when you are happy with your mother-in-law?* |
| *How can you tell when you are not happy with your mother-in-law?* |
| *Relationship with husband* |
| **Please tell me about your husband** |
| **Types of support** |
| **Have you ever had any problem and you family helped you?**   - **To solve the problem** - **To make you feel good about it?**   *If she doesn’t mention any problem:*  *Imagine you had a broken leg and needed to stay in bed. Still you need to carry grass for the animals. How would your family members help you to solve the problem and make you feel better about it?* |
| **What did your mother-in-law do to help you? What did she say?** |
| **What did/would your husband do to help you? What did/would he say?** |

Part 3 Free Exploration Social Support Water Carrying

**What do you think and how do you feel about carrying water alone or in a group?**

| *General Group Behavior* |
| --- |
| **Do you go alone or in a group?**   - *Why, why not?* - *Who goes with you?* |
| Support |
| **Does anyone except you carry water for your family?**   - *Any other person from your family?* - *Any other person outside your family? Why? Why not?* |
| **Did you ever help someone from another household to carry their water? Why? Why not?** |
| **Did you ever ask someone from another household for help to carry your water? Why did you need help? What did they say?** |
| **What would stop you from asking other people to help you carrying water?** |

Part 4a: Water Carrying during and after pregnancy Rating Task- Picture Task Social beliefs (randomize order)

1. This is Chenbagam, she delivered 2 weeks ago with her second child. She walks 30 minutes uphill and downhill per day, carrying water from the water scheme to her house.


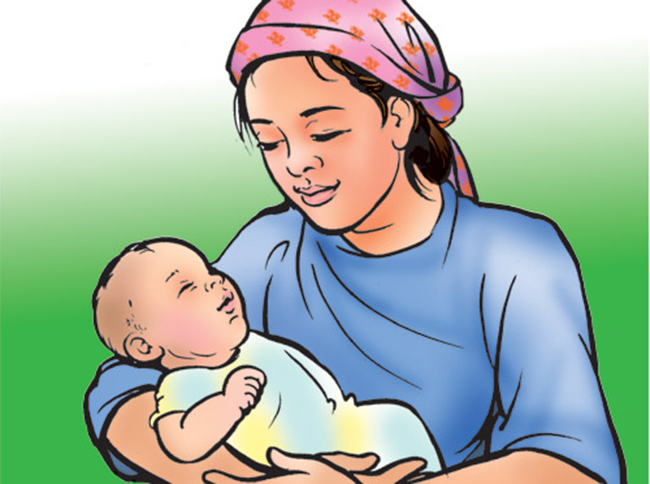


- What do you think, what kind of women is she? Why?
- What do other people think about her? Why?

*If participants perceives the behavior as something negative:*

- What can help her to improve the situation?
- What can she do?
- What can other people do?
- What can we ([blinded]Hospital) do?
- What would her mother-in-law do/ say to her? Why?
- What would her husband do/ say to her? Why?
- What do you think? How does she feel? Why?

1. This is Baijanthi, she delivered 2 weeks ago with her second child. She stays at home and does NOT carry water from the water scheme that is 30 minutes uphill and downhill from the house.


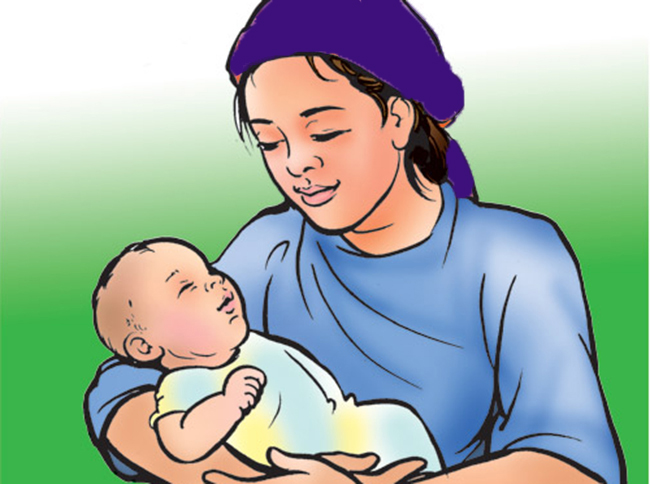


- What do you think, what kind of a woman is she? Why?
- What do other people think about her? Why?
- What would her mother-in-law do/ say to her? Why?
- What would her husband in law do/ say to her? Why?
- What do you think? How does she feel? Why?

Part 4b: Free Exploration Water Carrying in periods of pregnancy/childbirth

**Tell me about your daily routine during and in the three months after pregnancy**

| *Behavior* |
| --- |
| Do you change your working routine during and shortly after pregnancy?   - Why/ Why not? |
| **How was your Water Carrying routine during pregnancy?** |
| - *Whose decision was it that you carried water/ did not carry water?* - **Did you change the amount of water or the frequency?** - *Was it the same for all the months of pregnancy?*   *How did you feel about water carrying during pregnancy?* |
| **How was your Water Carrying routine in the three months after delivery?** |
| - **Whose decision was it that you carried water/ did not carry water?** - **Did you change the amount of water or the frequency?** - **Was it the same for all the months after delivery?**   *How did you feel about water carrying in the three months after delivery?* |
| *Knowledge* |
| **What are risks and benefits of carrying water during or after pregnancy?** |
| *Coping* |
| ***How does your household get water when you cannot go for water carrying because of pregnancy?*** |
| *Social support* |
| **How does your family behave when you are pregnant?** |
| *Does your family support you in water carrying?*   - *Which family member supports you?* - *What do they do to support you?* |
| *How does your husband behave when you are pregnant?* |
| *How do your in-laws behave when you are pregnant?* |
| **What will your family members say if you decide to stay at home during pregnancy instead of water carrying? What will they do?** |
| *Intervention acceptance: Not carrying during/ after pregnancy* |
| **What do you need to avoid carrying water and other heavy loads during and after pregnancy?** |
| *Intervention acceptance: Not carrying during/ after pregnancy – Social support* |
| - **Is there any person who can help you?** - **From within your family?**   - **How can this person be motivated to help you?** - **From your neighborhood?** - **If your neighbor woman carried loads for you when you were pregnant, would you also carry loads for her every time she is pregnant?** |
| *Intervention acceptance: Not carrying during/ after pregnancy – Structural support* |
| - **Is there anything you can buy by money that can help you to avoid carrying heavy loads during and after pregnancy?** - **Where can you get the money to get this?** |

**OPTIONAL**

Part 5 Free Exploration Risks Water Carrying

| *Security* |
| --- |
| **Are you talking to anyone on the way to water carrying? To whom?** |
| *If men:* **what are you talking about?** |

**Can you tell me a about risks and dangers for women when they carry water?**

| *Security* |
| --- |
| **Are you aware of any dangers women might face when they carry water? On the way**  **During night/ when it’s dark?** |
| **What do you think, what is the reason for these dangers?** |
| *Have you ever faced any danger when you went for water carrying?* |
| *Violence* |
| **What do you think about how risky it is for you to get verbally or physically attacked on your way to water carrying? Why?** |
| *The following question is about your personal experience, you do not need to answer if you don’t want to: Did anything like telling you bad things or hurting your body when water carrying ever happen to you?*  *If yes: Sometimes, people find it difficult to talk about unpleasant things that have happened to them. How do you feel telling me about this incident?* |
| *In case of telling about bad things she told/ done to her:*  **People do not have the right to do such things to you. I am very impressed how strong you are to live with this.**   - **What helps you to stay that strong and come over these events?** - **Is there anyone you can talk to, anyone you can trust?** |
| *What would you recommend to other women in the same situation, when someone says or does something bad to them?* |
| *Coping/ Protection* |
| **What needs to be changed to reduce these dangers on the way to water carrying?**   - **What can be done and by whom?** |
| *What can you do to feel security when you carry water? What would you recommend to other women who feel insecure?* |

Optional Part 6: Free Exploration Uterine Prolapse (Knowledge)

**Did you ever hear about uterine prolapse? Can you tell me about it?**

| *(Risk-)Knowledge* |
| --- |
| *Can you tell me about the reasons why women get uterine prolapse?* |
| *Knowledge on prevention* |
| **How can you avoid uterine prolapse?**  *If she says not carrying heavy loads/ any other reason:*   - ***How can you assure that you do/ don’t do this?*** |
| *Knowledge on treatment/ support* |
| **Which persons in a women’s life will help her when she has uterine prolapse?**   - **To feel better about your situation** - **To perform your daily tasks**   **To give your information on how to improve your situation?** |
| **What can women do if they have uterine prolapse?**   - **Who or what can help them?** - **Where can they go?** |
| **If they mention the hospital:**   - **Are there any alternative places to go besides the hospital?** - *If they mention: Alternative medicine:*   - Which steps did the people take?   - Which advice, concrete action did you and the people helping you take? - **How does it help you? How does it affect your body?** - **What about this option do you like more than going to a hospital?** - **What about this option do you like less than going to a hospital?** |
| **We have heard of women in this area having uterine prolapse but many of them do not come to visit the hospital.**  **What do you thing why?**  **What would prevent you from going to the hospital if you had uterine prolapse?** |
| **What can women with uterine prolapse do to prevent their condition from getting worse?** |
| *Intervention acceptance: Pelvic floor exercise* |
| - *Let us talk about the pelvic floor. Do you think the pelvic floor is important for women’s health? Why? Why not?* - **What can you do to strengthen your pelvic floor***?* - **Would you like to learn pelvic floor exercise if you knew how to do it?**    - **Why? Why not?** |
| *Intervention acceptance: Pessary ring* |
| **What kind of help would you like us (Dhulikhel Hospital) to provide to women with uterine?** |
| **Have you heard about the pessary ring?**  ***If she did not: It is a ring that will be inserted into the vagina to put back the uterus into the right place.***   - **Do you think it can help when you have uterine prolapse? Why/ why not?** - **How do you like it?**   **What would prevent you from using the pessary ring if you had uterine prolapse?** |

*Introduction: Sometimes, people find it difficult to talk about unpleasant things that have happened to them. How do you feel talking to me today about uterine prolapse?*

**For women without uterine prolapse only:**

| *Consequences* |
| --- |
| **Would anything in your life change if you had uterine prolapse?** |
| *Psychological Consequences* |
| **How do you feel when you think about that you have uterine prolapse?** |
| *Did anything in your mood change since you have uterine prolapse? What changed?* |
| *Social consequences/ Social support* |
| *Did your economic situation change since you suffer from uterine prolapse? Why?* |
| **Do other people know that you have uterine prolapse? How would they react if they knew?** |

**For women with uterine prolapse only:**

| *History* |
| --- |
| **When did you first notice that you had uterine prolapse and what did you do?** |
| **Can you tell me how life is with uterine prolapse?** |
| **Please tell me about it. How do you feel about this/these impact(s)?** |
| *Did anything in your life change since you have uterine prolapse?* |
| *Family Consequences/Domestic Violence* |
| **What does your mother-in-law say regarding the uterine prolapse?**   - *Does she know about your uterine prolapse?* - *Does she support you?* - *Does she say anything good or bad?* |
| Whom do you have in your life that supports you, or who you trust? |
| *The following questions refer to you and your husband. If you do not want to answer just give me a sign. You do not need to tell me why.*  **What does your husband say regarding your uterine prolapse?**   - *Does he know about your uterine prolapse?* - *If yes: How do you feel about that he knows?* - ***What does he do?*** - ***What does he say?***   ***Did his behavior towards you change since you have uterine prolapse?***  ***Did your behavior towards him change since you have uterine prolapse?*** |
| **Did your sex life change since you have uterine prolapse? How?**   - *Are you both satisfied with the amount and quality of sex?* - *Did you feel pain?*   - *Did you tell your husband? How did he react?* - *Do you feel mental stress?* |
| *In case of telling about forced sexual relation or any other incidents of physical/ emotional violence:*  *Sometimes, people find it difficult to talk about unpleasant things that have happened to them.* **How do you feel telling me about this incident?**   - - *How often did this happen?*   - *What helps you to stay that strong and come over these events?*   *Did you tell anybody about it? Why/ Why not?* |
| People do not have the right to do these things to you. I am very impressed how strong you are after this.   - What would you recommend to other women in the same situation, when someone says or does something bad to them? |

Uterine Prolapse only: Part 7: Free Exploration Quality of Health Services for Uterine Prolapse

What did you do to be helped when you suffered from uterine prolapse?

| *Accessibility* |
| --- |
| **Can you tell where you would go for help because of uterine prolapse?** |
| **Who took the decision that you went for health care?**   - *Can you choose to come there yourself, or did you need permission from somebody else? Who?* |
| *Are there times when you wanted to go to a health centre but couldn’t?*   - *Why?* |
| *Quality of health care* |
| **How were you helped?**   - - Which steps did the people helping you take to deal with your situation?   - Which advice, concrete action did you and the people helping you take?   - **Where there any advice or treatment they suggested that you refused? Why?** |
| **Did you receive the help you wanted?**  How satisfies have you been with the help you received? |
| **Did you receive any other help (alternative methods?** |

Part 8: Probing

1. Which questions were difficult for you to answer?
2. Is any questions irrelevant in your opinion?
3. Is any questions missing in your opinion?

Part 6: Socio-demographic questions

Finally, I have some personal questions. Again, we will keep this information strictly anonymous:

What is your ethnicity?

How old are you?

What are your household’s monthly expenses?

Are you in a relationship?

How many children do you have?

What is your highest education?

Name:

District:

Municipality:

Phone Number:

We recognise and commend your strength and courage, and thank you for taking the time to talk with us about your experiences. You have helped us a lot in understanding risks and benefits of water carrying. The information you shared with us will help to find strategies to support women in the field of water carrying, so that others may be helped by your experience.

Is there anything we did not mention yet you would like to add? Do you have any questions for us?

# **TABLE S5**: Sample characteristics and carrying behavior derived from qualitative interviews

|  | Woman in reproductive age (*n* = 12) | Mother-in-law (*n* = 5) | Husband *(n* = 4) |
| --- | --- | --- | --- |
| Age; (*Mean* (*SD*)) | 33.2(10.0) | 56.6 (8.7) | 49.5 (6.6) |
| Ethnicity^1^ ; (*frequency)* |  |  |  |
| Brahmin | 5 | 2 | 2 |
| Tamang | 1 | 1 | 1 |
| Newar | 1 |  |  |
| Chhetri | 2 | 1 |  |
| Dalit | 2 | 1 | 1 |
| Rai and Limbu | 1 |  |  |
| Education (*frequency)* |  |  |  |
| Illiterate | 2 | 4 | 2 |
| Informal education |  |  |  |
| Pre-primary | 2 | 1 |  |
| Primary passed | 2 |  |  |
| Lower secondary passed | 2 |  |  |
| Secondary | 4 |  | 1 |
| Higher secondary and above |  |  | 1 |
| Monthly expenses ;(*Mean* (*SD*)) | 20791 (13393) | 9900 (3664) | 21750 (102ß7) |
| Living conditions (*frequency)* |  |  |  |
| Single / living with children | 1 |  |  |
| Nuclear family | 4 |  | 2 |
| Joint family | 7 | 5 | 2 |
| Number of children; (Mean (SD)) | 2.3 (0.8) | 4.8 (3.6) | 3.3 (1.3) |
| Behavior during pregnancy (frequency) |  |  |  |
| Like usual | 7 | 5 |  |
| Carrying less amount | 4 |  |  |
| No carrying after second half | 1 |  |  |
| No carrying at all |  |  |  |
| Behavior 3 months postpartum (*frequency*) |  |  |  |
| Like usual / after less than 2 weeks |  | 3 |  |
| Carrying less amount up to 3 months |  | 1 |  |
| No carrying up to 2 weeks | 3 |  |  |
| No carrying up to 1 month | 2 |  |  |
| No carrying up to 2 months | 4 |  |  |
| No carrying up to 3 months | 3 | 1 |  |

^1^ Some women changed their ethnicity, ethnicity corresponds to ethnicity after marriage

**TABLE S6.** Additional quotations for qualitative themes.

| Theme | Quotation |
| --- | --- |
| Amount and frequency of carrying | “*I carried about 9 to 10 gagris [containers] of water because we had buffalos and cattle. I carried a 15-20 liter gagri in a doko [basket to carry loads].”* 9_wife: 143.  “*Did you carry that much water all throughout your pregnancy? R: Yes.”* 9_wife: 144 *– 145.* |
| **High risk perception but low personal vulnerability** | |
| Pain | “*It may cause back pain [to carry during pregnancy]. There would be no pros. Some people say that bleeding occurs if a woman carries water immediately after delivery. It didn’t happen to me.”* 5_daughterinlaw: 157. |
| Mental Health | *“[Carrying during and after pregnancy]* *might affect the baby and the mental condition of the woman.* 9_husband: 110 – 111*.* |
| Child’s Health | *“When they carry water at the waist it affects the baby greatly.”* 9_husband: 113. |
| Complications during pregnancy | *“They might also need a C-section [if they carry water during pregnancy].”* 5_motherinlaw: 143.  *“She fell down sometimes, and she had to worry when she was pregnant. She had to carry water when she was pregnant as well.”* 9_husband: 55. |
| Miscarriage | *“She might get a miscarriage [if she carries during pregnancy]; she may be weak.”* 5_motherinlaw: 143. |
| Uterine prolapse | *“When carrying heavy loads and doing a lot of hard chores for the woman who just delivered the baby there may be the risk of prolapse.”* 5_motherinlaw: 163.  *“I felt worried, but it was needed. I was worried that she might get uterine prolapse and bleeding; she might fall ill.”* 6_husband: 87. |
| Removal of uterus | *“If you carry heavy loads, then a uterus prolapse might happen and you need to operate, need to take out the uterus. There are also many people whose uterus has been removed, but I don’t have [that problem].”* 1_woman: 279. |
| Compensatory beliefs | *“During pregnancy, if you carry heavy loads, you shouldn’t squat during those times, you shouldn’t walk very fast.”* 9_wife: 198.  *“In my opinion, she [the women who carries water] is really healthy and maybe has no physical illness and pain. […] She should eat healthily and do oil massage and rest so she is safe.”* picture task carry 11_daughterinlaw: 111– 125. |
| **Negative and positive outcome expectancies for the avoidance of carrying** | |
| Less exercises | *When my wife had to carry water, she had many demerits but now that she doesn’t carry water, she has less exercise.”* 9_husband: 37. |
| Healthy child | ***“****Other people will say that you don’t have to carry water and you get the chance to rest so you can put on some weight and your child is healthy.”* picture task not carry 9_husband: 85.  **“***She [woman in picture task] doesn’t have to carry water, so it is nice. Her family will be healthy.”* picture task not carry 7_daughterinlaw: 152. |
| Low response efficacy | *“It would’ve been good if we didn’t have to work at all and could have enough rest. However, it [uterine prolapse] can happen to those who don’t work too.”* 8_motherinlaw: 189. |
| Karma beliefs | *“She [not carrying woman] might behave well or she might be from a good family. When a person is good, everybody will treat her well.”* picture task not carry 12_wife: 305.  “*Some say that it was God's decision [that they have uterine prolapse like my wife, so stay at home and don’t tell anyone about the problem.* 9_husband: 133. |
| Ability, not carrying when sick | *“When I was pregnant, I could carry water on my own, so he [husband] rarely carried.”* 12_wife: 179.  *“Did you carry the same amount for the whole nine months? - Only when I was sick I used to carry less.”* 8_motherinlaw: 134 – 135. |
| Affective Attitudes | *During past days I liked it [water-carrying], so I carried water. Now these days I don’t like carrying water, so I don’t carry: that’s it (laughing).”* 1_woman: 85. |
| Being greedy | *“My wife also carried water like this woman [in the picture task], so she has uterus prolapse. I always tell her she faced this prolapse not because of me but because of her greed to carry more water.”* picture task carry, 11_fatherinlaw: 69. |
| **Social influence and decision making** | |
| Carrying before marriage | ”*I used to carry when I was not married, when I was 12 or 13 years. It’s been seven years since I got married, but before that as well I used to carry loads.”* 2_woman: 82. |
| Family influence | *“She is not able to work but her family must also be like that (bad and culturally strict), if they were good then they would have cared about that women isn’t it? I think they are not a good family. I think they should have said ‘you are not able to earn money’, the pressure will also come from the home. [To interviewer] Isn’t it like that Miss? That’s human nature. It depends on the family.”* picture task carry 1_woman: 307. |
| Decision making control by family members | *“We all decided that she wouldn’t carry heavy loads [during pregnancy]. It’s bad to carry heavy loads during and after pregnancy, so my sisters didn’t allow her to carry heavy loads. My wife didn’t have to carry heavy loads for about 5 months.”* 9_husband: 104–105.  *“I decided not to make her [daughter-in-law] work [when she was pregnant].”* 10_motherinlaw: 160.  Contradictory quote: *“Everyone [in the family] decided that [to carry during pregnancy and shortly after delivery], but mostly me.”*12_wife: 161. |
| Hierarchical vs. shared decision making in families | “*Her husband may be in a dilemma whether to listen to his mother and let her work or to make his own decision and not let her work. He should also listen to the main person of the family. He might be following his mother.”* 12_wife: 137.  *“I decide that myself [whether my wife carries water during and after pregnancy]. My mother and father suggested me to support my wife and take care of her.”* 11_fatherinlaw: 108–109. |
| Family division of labor | “*It is easy if she [daughter-in-law] helps out. It is satisfying to have her here. She cleans, cooks and washes dishes, and lets me rest. This satisfies me. […] [Our relationship] is good. She does all the work and lets me rest.*” 7_motherinlaw: 63 – 67.  *“My eldest daughter-in-law lives here, and it’s easier. I carry less now.”* 5_motherinlaw: 27.  “*It got so much easier after she came. She carried all the loads*.” 7_motherinlaw: 35. |
| Descriptive norms | “*Everybody had to fetch water. Everybody had trouble due to water problems. If women woke up late, then they had to worry about not having enough water.”* 11­_fatherinlaw: 41.  *“Other people will think that it is normal [to carry after delivery]: ‘We also did all the work like her, and she should also do so.’ ”* picture task carry 12_wife: 133. |
| Injunctive norms | |
| Disapproval of resting | *“They [family members] might have behaved badly. They would have criticized me [for resting during pregnancy] and talked behind my back; compared me.”* 9_wife: 172 – 173.  *“[Other women might say] bad things. They [other women] might have said she’s sleeping and I need to bring water…’ (Laughing). … They must have said that the younger one [herself] is sitting and the elder one [sister in law] is coming to fetch the water.”* 3_woman: 190.  Contradictory quote: *“She [mother-in-law] is happy with whatever I do. She looks after my kids when I ‘m not around. I feel happy when she helps out. She’s happy with me.”* 5_daughterinlaw: 79. |
| Approval of carrying | *“She [mother-in-law] might think that her daughter-in-law has brought water from far away and she has taken good care of her and her family, so she might be good to her daughter-in-law. … She stays home and works really hard, so her husband may be happy with her. He [husband] must be reassured to have such a strong wife.”* picture task carry, 5_daughterinlaw: 111–117. |
| Disapproval of carrying | *“‘Why is she carrying water just after having delivered her baby?’ They [other people] say things like this. She shouldn’t carry water*.” picture task carry, 6_wife: 106. |
| Approval of resting | “*Other women must have said nice things to her [the woman who is resting after delivery]. She did a good job, and her body will also be good due to not working in the postpartum period.”* picture task not carry 2_woman: 215.  “*Others also told us that we should rest two months, so I did.”* 10_daugtherinlaw: 170. |
| Comply norms | *“The people said that we should carry loads when we’re pregnant, so I did.”* 12_wife: 179.  “*Poor mother-in-law, what she will say? During that time [in the past], the culture was like that. Everyone worked during that time, even they want to say don’t work if you can’t, the trend was like that (daughter-in-law needs to work at any phase of her life)”* 1_woman: 245.  Contradictory quote: *“They [in-laws] said things, but I rested anyway.”* 6_wife: 155 - 156 . |
| Caring husband | **“***He [husband] didn’t allow me to work when I was feeling unwell.”* 11_daughterinlaw: 177.  “*He [my son] also behaves well. He doesn’t hit her [daughter-in-law]; he tells her to rest and take care of her body and eat well. Tells her to do just the work she can.”* 5_motherinlaw: 151. |
| Mother-in -laws expectations towards daughter-in-law | “*Her mother-in-law might have scolded her: ‘I (mother-in-law) work and you’re resting, sitting, and eating’; must have said that. (Laughing). That’s what the mother-in-law says”* picture task carry 3_woman: 146. |
| Mother-in-law satisfaction when fulfilling work tasks | *“She’s done as much as she can. We can’t mistreat her, and we have been together and happy. She doesn’t complain and I don’t complain either.”* 5_motherinlaw: 67.  Contradictory quote: *“No matter how much we [daughters-in-law] help, she doesn’t count us.”* 11_daughterinlaw: 75. |
| Mother-in-law being aggressive | “*My mother-in-law complained about me to him [husband], and she also used to beat me.”* picture task carry 16_woman: 160. |
| **Making plans and overcoming barriers: Safe carrying techniques** | |
| Body posture | “*If people are carrying heavy loads, then their body should bend a little. If people are carrying fewer loads, then they should walk straight.”* 11_motherinlaw: 69. |
| Carrying less weight | “*One shouldn’t carry too much load or travel too long distances; should get much-needed rest.”* 8_motherinlaw: 163.  *”I think it is okay to carry between 5 and 10 liters.”* 11_daughterinlaw: 53.  “*Yes, it is possible [to carry small containers]. We can carry less water and make more trips.”* 12_wife: 84–85.  “*Did you carry that much water all throughout your pregnancy? R: Yes.”* 9_wife: 144–145. |
| Reduce frequency | *“I told her [daughter-in-law] to do less work. She brought water daily [during pregnancy]* *one or two times. [On normal days she carried] five to six times a day.* 5_motherinlaw: 135–137. |
| Use helping tools | *“What do you need to use safe carrying techniques?” -R: “I need ropes and doko [basket for the water container that is carried with straps on the forehead or shoulders] and sack.”* 5008_daugtherinlaw_5007: 80–81.  *“I cannot carry water on my waist. I use a doko to be safe.”* 8_daughterinlaw: 62–63.  “*In the past, people used to carry water in gagris [containers] on their waist, but now we carry it hanging in the hands ,and we use a doko for far away. I think it is healthy if we don’t carry water by putting loads on the waist or head or shoulder.”* 11_daughterinlaw: 51. |
| Ask for help | *“[The woman who carries after delivery can]may ask somebody like neighbors for help, saying she [is] unable to do it and she will help them [in return] when they are in need*.” picture task carry, 12_wife: 275. |
| Change lifestyle | *“We should learn other economic activities and stop rearing animals, so we won’t have to carry grass. We can earn money and bring home LPG gas and we won’t have to carry firewood.”* 12_wife: 81. |
| Pelvic floor exercise | “*Yes, I’ll learn it [pelvic floor exercise] and I’ll also teach it to others. … If something bad happens, then it becomes very hard for a woman. If I learn it, then maybe I can be healthy. I can also teach it to others. Even if it happens to me [something bad like uterine prolapse], I can make others aware. I am concerned about the new generation.”* 9_wife: 229–231. |
| **Lack of options and necessity to of carrying water** | |
| Lack of options | *“Even if I say I don’t like [carrying], nobody is going to help me. So for me, everything is good. If I say … I don’t want to work [still] no one is going to help me. And if I enjoy, then nobody is going to harm me. Does it make any difference what others think?”* 1_woman: 29*.* |
| *Obligation:* | *“I knew we shouldn’t carry heavy loads [during pregnancy] but due to compulsion I had to.”* 12_wife: 155. |
| Structural conditions | *“These days. I don’t need to go to the river and travel long. There are taps in the houses, so it’s easier now.”* 3_woman: 87.  *“These days they don’t carry water. The husband brings water for them by carrying it in his motorbike. There’s one house where there’s a pregnant lady, and the husband brings water for her.”* 3_woman: 176. |
| Sources not always reliable | *“Only when water doesn’t come from the tap do we go to fetch water. Otherwise, we don’t have to.”* 8_daughterinlaw: 19.  *“We used to get water from here, at the water spring. But that water spring [from where I used to collect water] is dry now.”* 1_woman: 111. |
| **Social support** | |
| Instrumental support family | *“When they were sick, then other people in the family carried water for them. There are mothers-in-law, fathers-in-law, and sisters-in-law who bring the water. So if somebody is ill, then they would not need to go to fetch water.”* 2_woman: 183. |
| Instrumental support others | *“The villagers won’t do anything for her. Perhaps one day one will bring food for her, the second day they will bring food, but they’ll never bring it every day. So, her own husband must do it or her mother-in-law or father in law must do it.”* picture task carry 15_woman: 240.  “*Yes [I help out my neighbors]. When they’re sick and unable and when no one is there.”* 5_motherinlaw: 92. |
| Informational support | *Doctors can give medicines and raise awareness. The husband and other family members can also suggest her to go for checkups.”* 10_motherinlaw: 191. |
| Emotional support | *“They (other people) should console her.”* picture task carry 9_wife: 115.  *“[After delivery] my mother came to support me and console me. I was young when I gave birth. The babies were 4 kg when delivered. My mother comforted me.”* 12_wife: 106–107. |
| Lack of instrumental support | *“Even when I’m not able to there's no one to carry for me.”* 6_wife: 43. |
| Support insufficient | *“When I was pregnant my husband rarely helped me. … The relatives came to help sometimes in the emergency time after I gave birth.”* 12_wife: 173–189. |
| Husband not always available | *“I didn’t let her carry heavy loads. I helped as much as I could when I was available, but in her postpregnancy period, she had to carry loads after about 2 to 3 months.”* 9_husband: 95. |
| Husband buys water or hires someone to help his wife. | “*Water is needed anyway, so I carry it in a big gagri [container]. Just 2 days ago, I brought water in a bus by paying a fare. Now it may be enough for 10-12 days, but after that we have to go and fetch water again.”* 6_husband: 17.  *“My husband helps out, and when my sons are home, they help. Some days ago, we even brought water in a truck due to a water shortage.”* 6_wife: 87.  *“I brought her mother and kept her at our home to help my wife after she gave birth.”* 9_husband: 96. |
| Mutual support husband wife | “*I carry more water then [when my wife is sick]. If I fall sick, then she carries all the water, and when she is ill, then I carry for her. The load shifts.”* 6_husband: 31. |
| Reasons to help or not mother-in-law | |
| Being weak / old | “*Mother-in-law and father-in -law already expired so who will do this? I need to do it myself. I have brothers-in-law, but they travelled abroad. So who will do it?”* 3_woman: 65.  “*I am weak now, so I can’t carry much [during the pregnancy and postpartum period of my daughter-in-law]. When I was stronger, I used to work, and it didn’t feel bad.*” 5_motherinlaw: 33. |
| Her personality | *“If her mother-in-law is nice, then she’ll say that she’ll look after her kids. If the mother-in-law isn't nice, then she’ll say that her daughter-in-law must carry water and do all the work herself.”* picture task carry 7_daughterinlaw: 136. |
| Relationship quality | *“As I’ve said, maybe the mother-in-law … has some discrimination-like feeling against her. She is her daughter-in-law and not her daughter, so she may behave rudely.”* picture task carry 11_daughterinlaw: 115. |
| Own past behavior | *“She might say that ‘I did it in my days and so should you. I worked even more, and you should work too’”*. picture task carry 12_husband: 79. |
| Traditional /modern mother-in-law | *“She must have a modern mother-in-law, so she may not say anything to her. (Laughing) Isn’t it? The traditional mother-in-law will say that they used to do this and that”*. picture task not carry 15_woman: 250. |
| Caring / loving as a reason for instrumental support | *"When the husband doesn’t care, then she must feel like she shouldn’t have married. Won’t she think like that? She’ll definitely think like that. He spends his life like that and I’m suffering. She must feel like that.”* picture task carry, 15_woman: 230.  *“If she [mother-in-law] loves her, then she’ll bring water by herself, won’t she?”* picture task carry 2_woman: 205. |
| Two different sides of mothers-in-law (being a resource or a threat to women’s health) | *“If her mother-in-law is nice, then she’ll say that she’ll look after her kids. If the mother-in-law isn't nice, then she’ll say that her daughter-in-law must carry water and do all the work herself.”* picture task carry 7_daughterinlaw: 136. |

# **S7.** SPSS SYNTAX to model two generalized estimating equations (GEE).

**Predictors of behavioral intention

GENLIN intend (REFERENCE=FIRST) WITH selfeff outex risk1 injnorm descnorm gen_ins_support age livingwithouthusband curr_pregnant delivered education SI_SES_b by eth mainsource_rainyseason

/MODEL risk1 outex selfeff injnorm descnorm gen_ins_support age SI_SES_b livingwithouthusband curr_pregnant delivered

education eth mainsource_rainyseason

INTERCEPT=YES

DISTRIBUTION=NORMAL LINK=IDENTITY

/CRITERIA METHOD=FISHER(1) SCALE=1 MAXITERATIONS=100 MAXSTEPHALVING=5 PCONVERGE=1E-006(ABSOLUTE)

SINGULAR=1E-012 ANALYSISTYPE=3(WALD) CILEVEL=95 LIKELIHOOD=FULL

/REPEATED SUBJECT=vdc_municipality WITHINSUBJECT=Participant_ID SORT=YES CORRTYPE=EXCHANGEABLE ADJUSTCORR=YES

COVB=ROBUST MAXITERATIONS=100 PCONVERGE=1e-006(ABSOLUTE) UPDATECORR=1

/MISSING CLASSMISSING=EXCLUDE

/PRINT CPS DESCRIPTIVES MODELINFO FIT SUMMARY SOLUTION (EXPONENTIATED) COVB.

*****Predictors of behavior

GENLIN safe_carry (REFERENCE=FIRST) WITH selfeff injnorm descnorm

intend gen_ins_support actionplanning_re coping_re age livingwithouthusband

education SI_SES_b curr_pregnant delivered by eth mainsource_rainyseason

/MODEL selfeff injnorm descnorm intend gen_ins_support actionplanning_re coping_re age SI_SES_b livingwithouthusband curr_pregnant delivered

education eth mainsource_rainyseason

INTERCEPT=YES

DISTRIBUTION=NORMAL LINK=IDENTITY

/CRITERIA METHOD=FISHER(1) SCALE=1 MAXITERATIONS=100 MAXSTEPHALVING=5 PCONVERGE=1E-006(ABSOLUTE)

SINGULAR=1E-012 ANALYSISTYPE=3(WALD) CILEVEL=95 LIKELIHOOD=FULL

/REPEATED SUBJECT=vdc_municipality WITHINSUBJECT=Participant_ID SORT=YES CORRTYPE=EXCHANGEABLE ADJUSTCORR=YES

COVB=ROBUST MAXITERATIONS=100 PCONVERGE=1e-006(ABSOLUTE) UPDATECORR=1

/MISSING CLASSMISSING=EXCLUDE

/PRINT CPS DESCRIPTIVES MODELINFO FIT SUMMARY SOLUTION (EXPONENTIATED) COVB.
